# Tuberculosis State Is Associated with Expression of Toll-Like Receptor 2 in Sputum Macrophages

**DOI:** 10.1128/mSphere.00475-17

**Published:** 2017-11-01

**Authors:** Karim Lakehal, David Levine, Kathleen F. Kerr, Pooja Vir, Natalie Bruiners, Alfred Lardizabal, Maria Laura Gennaro, Richard Pine

**Affiliations:** a Public Health Research Institute, New Jersey Medical School, Rutgers, The State University of New Jersey, Newark, New Jersey, USA; b Department of Biostatistics, University of Washington, Seattle, Washington, USA; c Global Tuberculosis Institute, New Jersey Medical School, Rutgers, The State University of New Jersey, Newark, New Jersey, USA; IIS/LAD/NIAID/NIH

**Keywords:** immunophenotyping, innate immunity, macrophage surface markers, pattern recognition receptors, tuberculosis biomarkers

## Abstract

During tuberculosis, macrophages are critical for both pathogen survival and host immune activation. Since expression of particular cell surface markers reflects cell function, we used flow cytometry to measure the abundance of surface markers associated with polarity, lipid uptake, or pattern recognition on macrophages found in induced sputum. Nine macrophage surface markers were examined from three groups of donors: infection-free, latent tuberculosis infection, and active pulmonary tuberculosis. Using a trend test, we found that expression of Toll-like receptor 2 was greater from absence of infection to latent infection and from latent infection to active tuberculosis. The results point to the possibility that innate immune cell phenotypes be used to distinguish among tuberculosis infection stages. Moreover, this study shows that readily accessible sputum macrophages have potential for tuberculosis diagnosis and prognosis.

**IMPORTANCE** *Mycobacterium tuberculosis* is an intracellular pathogen that parasitizes the host macrophage. While approximately two billion people are infected worldwide, only 5 to 10% become diseased with pulmonary tuberculosis, at least in the absence of comorbidities. Tuberculosis control requires development of noninvasive methods probing the host immune status to help distinguish latent infection from active tuberculosis. With such methods, high-risk individuals could be targeted for treatment before disease manifestation. Previous investigations have been based on examination of peripheral blood cells or, more rarely, lung macrophages obtained with invasive procedures, such as bronchoalveolar lavages. Here we show that differences exist in the expression of a surface protein (Toll-like receptor 2) between macrophages recovered from the sputum of individuals in different diagnostic groups: i.e., infection free, latent tuberculosis infection, and active pulmonary tuberculosis. Thus, phenotypic analysis of local macrophages obtained with noninvasive procedures can help distinguish among tuberculosis infection stages.

## Abstract (precis)

*Mycobacterium tuberculosis* is an intracellular pathogen that parasitizes the host macrophage. While approximately two billion people are infected worldwide, only 5 to 10% become diseased with pulmonary tuberculosis, at least in the absence of comorbidities. Tuberculosis control requires development of noninvasive methods probing the host immune status to help distinguish latent infection from active tuberculosis. With such methods, high-risk individuals could be targeted for treatment before disease manifestation. Previous investigations have been based on examination of peripheral blood cells or, more rarely, lung macrophages obtained with invasive procedures, such as bronchoalveolar lavages. Here we show that differences exist in the expression of a surface protein (Toll-like receptor 2) between macrophages recovered from the sputum of individuals in different diagnostic groups: i.e., infection free, latent tuberculosis infection, and active pulmonary tuberculosis. Thus, phenotypic analysis of local macrophages obtained with noninvasive procedures can help distinguish among tuberculosis infection stages.

## INTRODUCTION

Most of the 1.5 million annual deaths due to tuberculosis (TB) result from activation of latent *Mycobacterium tuberculosis* infection. Consequently, an important public health goal is to identify individuals who are progressing from latent infection to active disease before they become symptomatic and contagious. Existing diagnostics for *M. tuberculosis* infection, which rely on adaptive immune responses, such as delayed hypersensitivity or cytokine release by antigen-specific T cells (1), fail to meet this challenge (2). In contrast, recent transcriptomic analysis of peripheral blood cells points to innate immune cells as potential indicators of infection stage (3).

Among innate immune cells, macrophages are central in tuberculosis pathogenesis: these cells are parasitized by the pathogen, and they participate in establishing and maintaining chronic infection as well as in determining the immunopathology of active disease (4, 5). The remarkable functional plasticity of macrophages, which change phenotypes and functions in response to various environmental signals (6, 7), is often explored by monitoring expression of surface protein markers. Thus, it is conceivable that macrophage surface markers and the underlying phenotypes change with the spectrum of tuberculosis infection, presumably reflecting stage-specific microenvironments and cellular functions.

Multiple classes of surface protein markers have been used for macrophage phenotyping. For example, markers of macrophage polarization, which reflects the cell’s activation state, classify macrophages into two broad groups—the M1 and the M2 macrophages (6, 7). M1 macrophages participate in defense against intracellular pathogens (6, 8), including *M. tuberculosis* (5), while M2 cells likely create a favorable environment for intracellular microbial growth (9–11), due in part to reduced antimicrobial effector functions (12). Other macrophage responses to microbial infections are determined by protein receptors that recognize pathogen-associated molecular patterns (13). Expression of lipid receptors may also provide information about the functional state of macrophages during tuberculosis, since *M. tuberculosis* infection disrupts lipid homeostasis in macrophages (14–16). Thus, a variety of surface markers are available to characterize the relationship between macrophage functional phenotypes and tuberculosis state.

Another consideration for phenotyping efforts is the source of macrophages. In particular, studies of blood monocytes (3) are not preferred, because blood cells reflect systemic effects of infection rather than the lung environment where *M. tuberculosis* infection occurs (4). The respiratory locale can be examined by studying the macrophages from alveoli and lower airways; however, recovery of alveolar macrophages requires invasive bronchoalveolar lavage, which limits the availability of cells for analysis. A more suitable approach is to recover lower airway macrophages by sputum induction, a noninvasive, highly tolerable practice used for research and clinical management of several lung diseases, including tuberculosis (17–25). To date, such studies have not examined associations between immunophenotypes of macrophages found in sputum and tuberculosis infection state.

In the present study, we assessed the abundance of nine protein markers on the surface of sputum macrophages that are associated with macrophage polarization, pattern recognition, or lipid metabolism. We compared sputum macrophages from control subjects not latently infected with TB (LTBI−), latently infected subjects (LTBI+), and patients diagnosed with active pulmonary tuberculosis (PTB) to determine whether phenotypes of innate immune cells vary with tuberculosis infection stages. We find the abundance of Toll-like receptor 2 (TLR2) on the surface of sputum macrophages varies with tuberculosis infection stage.

## RESULTS

### Population and sample characteristics.

Ninety-four (61%) of 154 sputum samples yielded sufficient cell numbers (≥4 × 10^6^ total cells) for flow cytometry. The demographic characteristics of sputum donors (*n =* 94) are shown in Table S1 in the supplemental material. The key sputum characteristics were ~7 ± 2 ml of sample and ~(9 ± 5) × 10^6^ total cells, of which 45% ± 14% were macrophages (CD14 and CD11c double-positive cells).

10.1128/mSphere.00475-17.2 TABLE S1  Demographics of sputum donors. LTBI−, no tuberculosis infection; LTBI+, latent tuberculosis infection; PTB, active pulmonary tuberculosis. Download TABLE S1, DOCX file, 0.1 MB. Copyright © 2017 Lakehal et al. 2017 Lakehal et al. This content is distributed under the terms of the Creative Commons Attribution 4.0 International license.

### Expression of surface markers in sputum macrophages and tuberculosis state.

**FIG 1:**
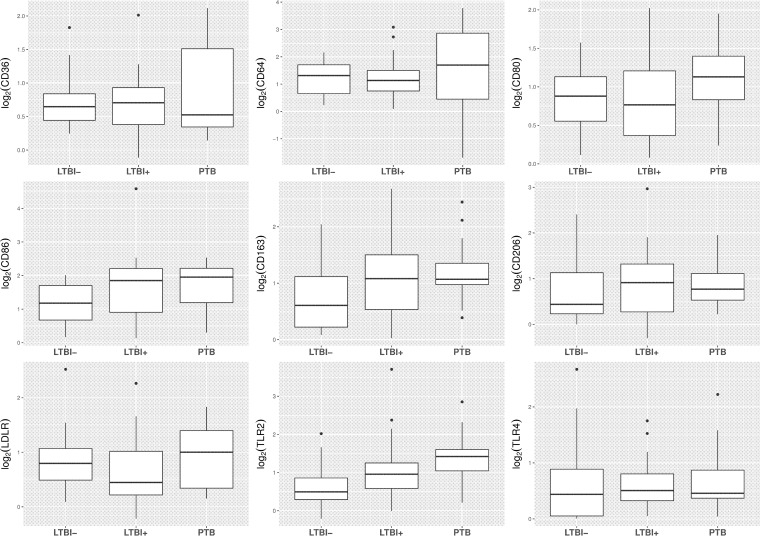
Expression levels of nine sputum macrophage markers by infection state. For each marker, the expression level was calculated as log_2_ of the ratio of the mean fluorescence intensity (MFI) of the marker to the MFI of an isotype-matched control antibody. Each panel represents one marker. The box plots show the first quartile, median, and third quartile of the distribution. The lower whisker extends from the first quartile to the smallest value of at most 1.5 × the interquartile range (IQR). The upper whisker extends from the third quartile to the largest value of at most 1.5 × IQR. Values exceeding the whisker limits are plotted individually. As shown in Table 2, the *P* value for trend was statistically significant only for TLR2 (*P* << 0.05); all other *P* values were >0.05. LTBI−, no tuberculosis infection; LTBI+, latent tuberculosis infection; PTB, active pulmonary tuberculosis.

To identify phenotypic differences among sputum macrophages obtained from subjects representing the different *M. tuberculosis* infection states (no latent TB infection [LTBI–], latent TB infection [LTBI+], and active pulmonary tuberculosis [PTB]), we measured the abundance of nine surface protein markers associated with macrophage function by flow cytometry (Table 1). We then investigated associations between surface marker levels and infection classification by linear regression analysis. We observed that the levels of several markers varied with infection state (Fig. 1 and column β in Table 2 [adjusted for donor’s age and plasma levels of C-reactive protein [CRP] as a control for nonspecific effects related to inflammation]). Only the trend for TLR2 expression was statistically significant (*P* < 0.001) (Table 2). Results were similar when the analysis was adjusted for CRP alone, donor’s age alone, or neither (data not shown). Association of TLR2 expression with severity of disease (expressed as extent of lung cavitation) was not statistically significant (*P* = 0.06) (see Table S2 in the supplemental material).

10.1128/mSphere.00475-17.3 TABLE S2  Test for trend of association of cavitation with sputum macrophage marker expression. The trend test was performed as in Table 1 on markers tested in at least 12 samples. n, number of observations; Beta, regression slope; SE, estimated standard error; P, *P* value. Download TABLE S2, DOCX file, 0.1 MB. Copyright © 2017 Lakehal et al. 2017 Lakehal et al. This content is distributed under the terms of the Creative Commons Attribution 4.0 International license.

**TABLE 1:** Surface protein markers evaluated in this study

Marker(s)	Function	Reference(s)
CD36	M2 marker, uptake of fatty acids and oxidized low-density lipoprotein, relevant to mycobacterial pathogenesis	36, 37
CD64	M1 marker (Fc-gamma receptor 1), binding to monomeric IgG-type antibodies, macrophage activation	38
CD80, CD86	M1 markers, costimulatory signals necessary for T cell activation and survival	39
CD163	M2 marker, macrophage iron uptake, relevant to mycobacterial pathogenesis	40
CD206	Pattern recognition receptor (mannose receptor), *M. tuberculosis* uptake	41, 42
LDLR	Cell surface receptor, cholesterol homeostasis	43
TLR2, TLR4	Pattern recognition receptors, interaction with *M. tuberculosis* lipoproteins and lipopolysaccharides	13, 41

**TABLE 2:** Test for trend of association of infection state with sputum macrophage marker expression^a^

Marker	*n*	β	SE	*P* value
CD36	34	0.23	0.19	0.2348
CD64	44	0.12	0.31	0.7009
CD80	46	0.08	0.12	0.4952
CD86	46	0.23	0.16	0.1612
CD163	45	0.19	0.14	0.1811
CD206	89	0.06	0.10	0.5698
LDLR	43	0.03	0.16	0.8467
TLR2	91	0.30	0.09	0.0009
TLR4	45	0.15	0.17	0.3734

^a^ The expression level of each marker was calculated as described in the legend to Fig. 1. The test for trend is described in Materials and Methods. *n*, number of observations; β, regression slope; SE, estimated standard error.

We conclude that the level of TLR2 (but not TLR4) changes with tuberculosis infection state but not with tuberculosis disease severity. The results do not indicate that macrophage polarity or the expression of the other selected markers tested distinguishes among tuberculosis infection states.

## DISCUSSION

Immunophenotyping of sputum macrophages from three tuberculosis diagnostic groups showed that (i) macrophages in sputum expressed both M1 and M2 polarization markers, thus recapitulating the simultaneous presence of M1 and M2 phenotypes of alveolar and granuloma macrophages in pulmonary tuberculosis (26–29), and (ii) among the markers tested, only TLR2 exhibited a statistically significant trend of increased abundance across the three diagnostic groups. This result is consistent with the elevated level of TLR2 gene expression in blood cells and in sputum cells from pulmonary tuberculosis patients relative to control subjects (3, 30). While a marker or markers that can conclusively distinguish between active tuberculosis and latent infection remain to be identified, our study unequivocally demonstrates that (i) differences exist in the expression of innate immune markers in relation to tuberculosis state, and (ii) easily obtained sputum macrophages can be used to detect those differences.

Our work advances immunodiagnostic tuberculosis research on multiple fronts. First, by providing the first evidence for an association between immunophenotypes of macrophages found in sputum and tuberculosis infection state, the present study identifies innate immune cell phenotyping as a promising biomarker discovery space. In contrast, much of tuberculosis biomarker discovery has previously focused largely on adaptive (T cell) immune responses (31), with overall disappointing results (2, 32, 33), despite indications that myeloid cell signatures in blood differ between active tuberculosis and latent infection (34). Second, our approach shows the value of characterizing cells in sputum, while previous sputum investigations of pulmonary disease, including tuberculosis, addressed cell differentials and detection of fluid-phase inflammatory and immunomodulatory molecules (17–25). Finally, even when sputum cells (but not specifically macrophages) were characterized for expression of genes associated with innate and adaptive immune responses associated with tuberculosis (30), donors having latent tuberculosis infection with normal chest X rays, who represent the largest reservoir of *M. tuberculosis* infection (35), were not included in the study. Our study strongly supports the possibility that characterization of readily obtainable sputum macrophages for surface protein markers that can be detected by clinical flow cytometry constitutes a novel approach for identification of infection state markers for tuberculosis diagnosis and prognosis.

## MATERIALS AND METHODS

### Donor recruitment.

Participants were recruited from two regional chest clinics in Essex and Middlesex Counties, New Jersey. All participants provided informed consent as approved by the Rutgers University Institutional Review Board.

### Clinical definitions of tuberculosis state.

Active pulmonary tuberculosis (PTB) was defined based on symptoms suggestive of active tuberculosis (i.e., cough for more than 2 weeks, fever, night sweats, and weight loss) and/or an abnormal chest radiograph consistent with PTB. Diagnosis was confirmed by *M. tuberculosis* culture-positive sputum, regardless of initial sputum smear findings (i.e., presence/absence of acid-fast bacilli). Latent *M. tuberculosis* infection (LTBI+) was defined by lack of symptoms, a negative chest radiograph, and a positive tuberculin skin test (induration of ≥10 mm) or interferon gamma release assay with QuantiFERON-TB Gold (according to the manufacturer’s guidelines). Absence of *M. tuberculosis* infection (LTBI−) was defined by a negative tuberculin skin test or interferon gamma release assay. Participants with PTB were enrolled within 8 weeks after initiation of antituberculosis treatment. All donors were free from HIV-1 infection.

PTB disease severity was defined by examining chest radiographs and determining the extent of lung cavitation, which was measured at the point of maximum diameter in 6-ft posteroanterior chest X-ray films. Patients were grouped as follows: 1, absence of cavitation; 2, presence of single or multiple cavities with a diameter of <4 cm in aggregate; or 3, presence of single or multiple cavities with a diameter of ≥4 cm in aggregate.

### Plasma levels of CRP.

CRP measurements were obtained by using particle-enhanced immunoturbidimetric assays and Roche Cobas c501 analyzers.

### Induced sputum.

To minimize contamination from the oral cavity, participants were asked to brush, rinse, and gargle with tap water until the returned fluid was free of debris. An SU99 ultrasonic nebulizer (WestPrime Health Care, Chino, CA) was used to produce a mist of 3% saline solution that patients were instructed to deeply inhale and exhale through the mouth. Inhalation was interrupted each time the patient expectorated sputum into a sterile container; inhalation was resumed until ~10 ml of sputum was obtained or until a maximum of 20 min of inhalation was reached. Sputum samples were stored at 4°C for ≤2 h, diluted with 1 volume of phosphate-buffered saline (PBS) at pH 7.4 and 0.1 volume of 100 mM dithiothreitol, shaken for 10 min on a platform rocker, gravity filtered twice through a 70-μm-pore nylon strainer, and centrifuged for 10 min at 450 × *g* at 4°C. Cells were washed twice and resuspended in PBS; the total cell number was determined using a Neubauer hemocytometer. Cells were centrifuged again and resuspended in PBS plus 1% bovine serum albumin at a density of 2.5 × 10^6^ cells/ml for antibody staining. All procedures were approved by the Rutgers University Institutional Biosafety Committee.

### Measurement of macrophage cell surface markers by flow cytometry.

Sputum cells (1 × 10^6^) were incubated for 30 min on ice with specific mouse anti-human monoclonal antibodies or isotype-matched negative-control antibodies. The following antibodies were used: anti-CD14–Alexa 700 clone M5E2, anti-CD11c–phycoerythrin (PE)–CY7 clone B-LY6, anti-TLR2–fluorescein isothiocyanate (FITC) clone TL2.1, anti-CD163–PE clone GHI/61, and anti-TLR4–allophycocyanin (APC) clone HTA125 from Becton, Dickinson, San Jose, CA; anti-CD80–FITC clone 2D10.4, anti-CD86–PE clone IT2.2, anti-CD206–Efluor450 clone 19.2, anti-CD64–Pacific Blue clone 10.1, anti-CD36–APC/CY7 clone 5-271, isotype control mouse IgG2a APC/Cy7 clone MOPC-173, and mouse IgG1 Pacific Blue clone MOPC-21 from BioLegend, San Diego, CA; anti-low-density lipoprotein receptor (LDLR)–APC clone 472413 from R&D, Minneapolis, MN; and isotype control mouse IgG1 (FITC, APC, or eFluor450 conjugated) clone P3.6.2.8.1 from EBioscience, San Diego, CA. After incubation with antibody, cells were washed twice with phosphate-buffered saline and fixed in 4% paraformaldehyde for 30 min. A total of 100,000 events per sample was recorded with a Becton, Dickinson LSR II flow cytometer, which was calibrated with Calibrite beads (Becton, Dickinson, San Jose, CA). Data were analyzed by FlowJo Software v.7.6.5 (FlowJo, LLC, Ashland, OR).

Identification of macrophages in all samples was based on forward and side light scattering and on positivity for both CD14 and CD11c macrophage markers. Experimental markers were tested in two separate panels to accommodate the numbers of macrophages in the samples and fluorophore panels. Panel 1 (CD80, CD86, CD163, TLR2, and CD206) was tested on the initial ~50 donors. A preliminary analysis identified markers in panel 1 that failed to distinguish among groups; testing of these (CD80, CD86, and CD163) was discontinued. Panel 2 (TLR2, CD206, LDLR, CD36, CD64, and TLR4) was tested on an additional ~40 donors. For each experimental marker, the expression level was calculated as the ratio of the mean fluorescence intensity (MFI) of the marker to the MFI of an isotype-matched control antibody.

### Statistical analysis.

Two sets of linear regression analyses were performed. One model was used to estimate the association between macrophage marker levels and infection classification. We performed a linear regression of log_2_ of the marker level (*M*) versus the infection state (*S*) coded as 1, 2, and 3 (LTBI−, LTBI+, and PTB, respectively [see “Clinical definitions of tuberculosis state”]): log_2_ *M* = β_0_ + β_1_ *S* + β_2_ CRP + β_3_ age. We assessed the evidence of a trend by testing the null hypothesis β_1_ = 0, where β_1_ represents the CRP- and age-adjusted difference in mean log_2_ *M*, on average, for groups differing by one step in infection state. The outcome of the analysis was left unadjusted or was adjusted for (i) level of C-reactive protein (CRP) in the subject’s blood at the time of sputum collection, which served to control for nonspecific effects related to inflammation, and/or (ii) donor’s age, which controlled for the generally younger age of LTBI− participants compared to the other participants. CRP values and donor’s age are shown in Fig. S1 in the supplemental material. A second linear regression model was used to estimate the association between macrophage marker levels in PTB patients and disease severity: log_2_ *M* = β_0_ + β_1_ *V* + β_2_ CRP + β_3_ age, where *V* is the disease severity category (cavitation grade) coded 1, 2, or 3 (0, <4, and >4 mm, respectively [see “Clinical definitions of tuberculosis state”]). Associations between marker levels and disease severity were adjusted for age and CRP. All statistical tests were Wald tests, and robust standard error estimates were used. The threshold for statistical significance was set at 0.05.

10.1128/mSphere.00475-17.1 FIG S1  Distribution of analysis covariates by infection state. The box plots show the distribution of C-reactive protein (CRP) (milligrams per liter [left panel]) and age (years [right panel]) of the study participants grouped by infection state. See the legend to Fig. 1 for interpretation of box plots. LTBI−, no tuberculosis infection; LTBI+, latent tuberculosis infection; PTB, active pulmonary tuberculosis. Download FIG S1, TIF file, 0.7 MB. Copyright © 2017 Lakehal et al. 2017 Lakehal et al. This content is distributed under the terms of the Creative Commons Attribution 4.0 International license.
