# Supplementary material for: Tuberculosis State Is Associated with Expression of Toll-Like Receptor 2 in Sputum Macrophages
Source: mSphere. 2017 Nov 1;2(6):e00475-17. doi: 10.1128/mSphere.00475-17 (PMC5663984; doi:10.1128/mSphere.00475-17)
Supplement: TABLE S1 [file sph006172397st2.docx]

| Donor status |  | LTBI- (n=28) | LTBI+ (n=45) | PTB (n=22) |
| --- | --- | --- | --- | --- |
| Gender | Male | 14 | 17 | 13 |
|  | Female | 14 | 28 | 9 |
| Age (years) | 18-30 | 16 | 7 | 4 |
|  | 30-40 | 4 | 14 | 5 |
|  | 40-50 | 3 | 10 | 6 |
|  | 50-60 | 4 | 6 | 2 |
|  | >60 | 1 | 8 | 5 |
| Race | Asian | 8 | 7 | 2 |
|  | Black | 6 | 14 | 8 |
|  | Caucasian | 14 | 24 | 12 |
| Place of Birth | U.S.A. | 15 | 9 | 4 |
|  | Central/South America | 6 | 26 | 15 |
|  | Africa | 1 | 3 | 1 |
|  | Asia | 6 | 6 | 2 |
|  | Europe | 0 | 1 | 0 |
