# Supplementary material for: Tuberculosis State Is Associated with Expression of Toll-Like Receptor 2 in Sputum Macrophages
Source: mSphere. 2017 Nov 1;2(6):e00475-17. doi: 10.1128/mSphere.00475-17 (PMC5663984; doi:10.1128/mSphere.00475-17)
Supplement: TABLE S2 [file sph006172397st3.docx]

| Marker | n | Beta | SE | P |
| --- | --- | --- | --- | --- |
| CD80 | 12 | -0.09 | 0.22 | 0.6873 |
| CD86 | 12 | -0.08 | 0.32 | 0.8173 |
| CD163 | 12 | 0.20 | 0.23 | 0.4091 |
| CD206 | 17 | -0.16 | 0.17 | 0.3431 |
| TLR2 | 19 | -0.35 | 0.17 | 0.0643 |
